# COVID-19 Vaccination Intent, Perceptions, and Reasons for Not Vaccinating Among Groups Prioritized for Early Vaccination — United States, September and December 2020

**DOI:** 10.15585/mmwr.mm7006e3

**Published:** 2021-02-12

**Authors:** Kimberly H. Nguyen, Anup Srivastav, Hilda Razzaghi, Walter Williams, Megan C. Lindley, Cynthia Jorgensen, Neetu Abad, James A. Singleton

**Affiliations:** ^1^National Center for Immunization and Respiratory Diseases, CDC; ^2^Leidos, Inc., Atlanta, Georgia; ^3^Center for Global Health, CDC.

*On February 9, 2021, this report was posted online as an *MMWR *Early Release.*

As of February 8, 2021, 59.3 million doses of vaccines to prevent coronavirus disease 2019 (COVID-19) had been distributed in the United States, and 31.6 million persons had received at least 1 dose of the COVID-19 vaccine (*1*). However, national polls conducted before vaccine distribution began suggested that many persons were hesitant to receive COVID-19 vaccination (*2*). To examine perceptions toward COVID-19 vaccine and intentions to be vaccinated, in September and December 2020, CDC conducted household panel surveys among a representative sample of U.S. adults. From September to December, vaccination intent (defined as being absolutely certain or very likely to be vaccinated) increased overall (from 39.4% to 49.1%); the largest increase occurred among adults aged ≥65 years. If defined as being absolutely certain, very likely, or somewhat likely to be vaccinated, vaccination intent increased overall from September (61.9%) to December (68.0%). Vaccination nonintent (defined as not intending to receive a COVID-19 vaccination) decreased among all adults (from 38.1% to 32.1%) and among most sociodemographic groups. Younger adults, women, non-Hispanic Black (Black) persons, adults living in nonmetropolitan areas, and adults with lower educational attainment, with lower income, and without health insurance were most likely to report lack of intent to receive COVID-19 vaccine. Intent to receive COVID-19 vaccine increased among adults aged ≥65 years by 17.1 percentage points (from 49.1% to 66.2%), among essential workers by 8.8 points (from 37.1% to 45.9%), and among adults aged 18–64 years with underlying medical conditions by 5.3 points (from 36.5% to 41.8%). Although confidence in COVID-19 vaccines increased during September–December 2020 in the United States, additional efforts to tailor messages and implement strategies to further increase the public’s confidence, overall and within specific subpopulations, are needed. Ensuring high and equitable vaccination coverage across all populations is important to prevent the spread of COVID-19 and mitigate the impact of the pandemic.

The Advisory Committee on Immunization Practices (ACIP) has issued interim recommendations for COVID-19 vaccine allocation, with initial limited supplies of vaccines recommended for health care personnel and residents of long-term care facilities (phase 1a); frontline essential workers and persons aged ≥75 years (phase 1b); and persons aged 65–74 years, persons aged 16–64 years at high risk for severe COVID-19 illness because of underlying medical conditions,* and other workers in essential and critical infrastructure sectors^†^ not included in phases 1a and 1b (phase 1c) (*3*,*4*). Vaccinating a large proportion of persons in the United States against COVID-19 is critical for preventing SARS-CoV-2–associated morbidity and mortality and helping bring an end to the global pandemic.

During September 3–October 1, CDC conducted a probability-based Internet panel survey (IPSOS KnowledgePanel)^§^ of a nationally representative sample of 3,541 U.S. adult panelists aged ≥18 years to assess intent to receive a COVID-19 vaccine and perceptions about the vaccine (*5*). During December 18–20, CDC sponsored questions on two probability-based household panel omnibus surveys (IPSOS KnowledgePanel^¶^ and NORC Amerispeak**) administered to 2,033 panelists (approximately 1,000 panelists each) to reassess COVID-19 vaccination intent and related perceptions.^††^ This activity was reviewed by CDC and was conducted consistent with applicable federal law and CDC policy.^§§^ The same questions about COVID-19 vaccine intentions, perceptions, and reasons for not receiving a COVID-19 vaccine were asked in the September and December surveys. However, most respondents were different for each survey; only 123 panelists (3.5%) completed both the September and December IPSOS survey. Intent was assessed by response to the following question: “If a vaccine against COVID-19 were available today at no cost, how likely would you be to get it?” Response options were “absolutely certain,” “very likely,” “somewhat likely,” and “not likely.” Respondents who answered “absolutely certain” or “very likely” to receive a COVID-19 vaccination were defined as intending to be vaccinated, and respondents who answered “not likely” were defined as not intending to be vaccinated. Vaccination intentions and related perceptions were stratified by the following three mutually exclusive groups representing the ACIP priorities for initial doses of COVID-19 vaccine after health care providers and long-term care residents: 1) essential workers,^¶¶^ 2) adults aged 18–64 years with underlying medical conditions, and 3) adults aged ≥65 years.*** Sample size for the December surveys was not large enough to stratify the analysis by age group (65–74 years versus ≥75 years) or essential worker subgroups (health care personnel, other frontline essential workers, and other non-frontline essential workers). Analyses were also conducted to provide estimates among all adults and among adults not included in the initial ACIP priority groups (aged 18–64 years with no underlying medical conditions and who were not essential workers). Responses to questions on intent, perceptions, and reasons for not getting vaccinated were examined by sociodemographic characteristics and priority groups for the September and December surveys. Because of similar sampling methods and characteristics of respondents, the averages of the estimates from the two December surveys were calculated, and the difference between the September survey and the average of the December surveys was determined using t-tests. All surveys were weighted to ensure representativeness of the U.S. population, and all analyses were conducted using SAS-callable SUDAAN (version 11.0; RTI International).

From September to December, the proportion of adults reporting intent to receive COVID-19 vaccine as absolutely certain or very likely increased significantly by 9.7 percentage points (from 39.4% to 49.1%), and the proportion reporting nonintent decreased by 6.0 percentage points (from 38.1% to 32.1%) (Table 1). Among priority groups, intent increased by 17.1 percentage points among adults aged ≥65 years (from 49.1% to 66.2%), by 8.8 percentage points among essential workers (from 37.1% to 45.9%), and by 5.3 percentage points among adults aged 18–64 years with underlying medical conditions (from 36.5% to 41.8%) (Supplementary Figure, https://stacks.cdc.gov/view/cdc/101583).

**TABLE 1:** COVID-19 vaccination intent among surveyed adults, by vaccination priority group — United States, September and December 2020

Characteristic	Weighted % (95% CI)				
	IPSOS, Sep 2020* (n = 3,541)	IPSOS, Dec 2020^†^ (n = 1,005)	NORC, Dec 2020^§^ (n = 1,028)	Average of Dec IPSOS^†^ and NORC^§^ estimates (n = 2,033)	Difference between Dec and Sep estimates^¶^
**All adults**					
Intent to get COVID-19 vaccine					
Absolutely certain/Very likely**	39.4 (37.7 to 41.2)	50.3 (46.9 to 53.6)	47.8 (42.7 to 52.8)	49.1 (46.0 to 52.1)	9.7 (6.2 to 13.2)
Somewhat likely	22.5 (21.0 to 24.0)	16.8 (14.2 to 19.4)	21.0 (17.4 to 24.8)	18.9 (16.4 to 21.4)	−3.6 (−6.5 to −0.7)
Not likely	38.1 (36.4 to 39.8)	33.0 (29.7 to 36.2)	31.2 (26.5 to 35.8)	32.1 (29.6 to 34.6)	−6.0 (−9.0 to −3.0)
**Essential workers**					
Intent to get COVID-19 vaccine					
Absolutely certain/Very likely**	37.1 (34.2 to 40.0)	49.0 (42.9 to 55.1)	42.8 (34.9 to 50.6)	45.9 (40.9 to 50.9)	8.8 (3.0 to 14.6)
Somewhat likely	22.8 (20.2 to 25.3)	14.4 (9.9 to 19.1)	23.0 (16.6 to 29.6)	18.7 (14.0 to 23.4)	−4.1 (−9.4 to 1.2)
Not likely	40.2 (37.3 to 43.2)	36.6 (30.7 to 42.3)	34.2 (25.8 to 42.6)	35.4 (30.8 to 40.0)	−4.8 (−10.3 to 0.7)
**Adults aged ≥65 yrs**					
Intent to get COVID-19 vaccine					
Absolutely certain/Very likely**	49.1 (45.6 to 52.6)	66.5 (60.0 to 73.0)	65.8 (59.0 to 72.6)	66.2 (61.5 to 70.8)	17.1 (11.3 to 22.9)
Somewhat likely	21.1 (18.3 to 23.9)	12.8 (8.4 to 17.2)	17.4 (12.0 to 22.9)	15.1 (11.6 to 18.6)	−6.0 (−10.5 to −1.5)
Not likely	29.8 (26.6 to 33.0)	20.6 (14.9 to 26.4)	16.8 (10.2 to 23.3)	18.7 (14.3 to 23.0)	−11.1 (−16.5 to −5.7)
**Adults aged 18–64 yrs with underlying medical conditions**					
Intent to get COVID-19 vaccine					
Absolutely certain/Very likely**	36.5 (33.4 to 39.6)	44.8 (38.0 to 51.5)	38.8 (32.6 to 45.1)	41.8 (37.2 to 46.4)	5.3 (−0.2 to 10.8)
Somewhat likely	23.0 (20.3 to 25.7)	19.2 (13.3 to 25.0)	20.6 (14.7 to 26.6)	19.9 (15.7 to 24.1)	−3.1 (−8.1 to 1.9)
Not likely	40.4 (37.3 to 43.7)	36.0 (29.4 to 42.8)	40.5 (34.5 to 46.5)	38.3 (33.8 to 42.8)	−2.1 (−7.6 to 3.4)
**Adults aged 18–64 yrs without underlying medical conditions and nonessential workers**					
Intent to get COVID-19 vaccine					
Absolutely certain/Very likely**	38.0 (34.5 to 41.4)	46.3 (40.5 to 52.1)	48.7 (40.0 to 57.4)	47.5 (42.3 to 52.7)	9.5 (3.3 to 15.7)
Somewhat likely	22.4 (19.4 to 25.2)	18.4 (13.8 to 23.1)	22.2 (13.2 to 31.3)	20.3 (15.2 to 25.4)	−2.1 (−8.0 to 3.8)
Not likely	39.8 (36.4 to 43.1)	35.2 (29.5 to 41.0)	29.0 (20.9 to 37.2)	32.2 (27.2 to 37.1)	−7.6 (−13.6 to −1.6)

**Abbreviations:** CI = confidence interval; COVID-19 = coronavirus disease 2019.

* IPSOS KnowledgePanel Survey, fielded September 3–October 1.

^†^ IPSOS KnowledgePanel Omnibus Survey, fielded December 18–20.

^§^ NORC AmeriSpeak Omnibus Survey, fielded December 18–20.

^¶^ CIs for differences that exclude zero are statistically significant.

** Might include some persons who already received the COVID-19 vaccine.

Vaccination nonintent differed by sociodemographic characteristics and decreased across most socioeconomic groups from September to December (Table 2). For example, nonintent decreased by 10.3 percentage points among adults aged 50–64 years and by 11.1 percentage points among adults aged ≥65 years. Although nonintent was higher among women, nonintent among both women and men decreased by 6.0 percentage points between September and December. Nonintent was highest among Black persons in September (56.1%) and December (46.5%) compared with other racial/ethnic groups, with the difference between months (−9.6) not statistically significant. Nonintent was higher among adults with lower educational attainment and lower income but decreased across most education and income categories: among adults with a high school diploma or less, nonintent decreased 7.9 percentage points, and in households with annual incomes of $35,000–$49,999, nonintent decreased by 10.8 percentage points. Vaccination nonintent also decreased in metropolitan statistical areas^†††^ by 6.7 percentage points and among adults in all regions of the United States, except the Northeast, including decreases of 8.3 percentage points in the South, 6.8 in the Midwest, and 6.8 in the West. In December, nonintent was highest among persons without health insurance (44.5%), compared with those who had private health insurance (30.7%) and public health insurance (29.6%), and was similar in September and December.

**TABLE 2:** Prevalence of intent not to receive COVID-19 vaccine, by selected characteristics — United States, September and December 2020

Characteristic	Weighted % (95% CI)		
	IPSOS, Sep 2020* (n = 3,541)	Average of Dec IPSOS^†^ and NORC^§^ estimates (n = 2,033)	Difference between Dec and Sep estimates^¶^
**All adults, aged ≥18 yrs**			
**Age group, yrs**			
18–49 (ref)	39.5 (36.9 to 42.0)	37.6 (33.5 to 41.7)	−1.9 (−6.7 to 3.0)
50–64	42.0 (38.9 to 45.2)	31.7 (26.6 to 36.8)	−10.3 (−16.3 to −4.3)
≥65	29.8** (26.6 to 33.0)	18.7** (14.3 to 23.1)	−11.1 (−16.5 to −5.7)
**Sex**			
Male	33.8** (31.4 to 36.2)	27.8** (24.7 to 30.9)	−6.0 (−9.9 to −2.1)
Female (ref)	42.1 (39.7 to 44.6)	36.0 (31.4 to 40.6)	−6.1 (−11.3 to −0.9)
**Race/Ethnicity**			
White, non-Hispanic (ref)	35.9 (33.8 to 38.1)	30.3 (27.4 to 33.2)	−5.6 (−9.2 to −2.0)
Black, non-Hispanic	56.1** (51.4 to 60.8)	46.5** (36.8 to 56.2)	−9.6 (−20.4 to 1.2)
Hispanic	36.4 (31.8 to 41.0)	32.4 (26.2 to 38.6)	−4.0 (−11.7 to 3.7)
Other/Multiple races, non-Hispanic	32.1 (27.4 to 36.8)	24.4 (17.0 to 31.9)	−7.7 (−16.5 to 1.1)
**Educational status**			
High school or less (ref)	47.0 (44.0 to 50.0)	39.1 (34.0 to 44.2)	−7.9 (−13.8 to −2.0)
Some college or college graduate	35.8** (33.4 to 38.2)	30.9** (27.9 to 33.8)	−4.9 (−8.7 to −1.1)
Above college graduate	23.8** (20.3 to 27.3)	15.7** (11.1 to 20.4)	−8.1 (−13.9 to −2.3)
**Employment status**			
Employed (ref)	38.6 (36.5 to 40.8)	32.3 (29.2 to 35.4)	−6.3 (−10.1 to −2.5)
Not employed/Not in workforce	36.6 (33.8 to 39.5)	31.5 (27.1 to 35.9)	−5.1 (−10.3 to 0.1)
**Annual household income, $**			
<35,000 (ref)	44.0 (40.2 to 47.7)	38.3 (32.4– to 44.1)	−5.7 (−12.6 to 1.2)
35,000–49,999	45.1 (40.0 to 50.2)	34.3 (26.7 to 41.9)	−10.8 (−20.0 to −1.6)
50,000–74,999	39.8 (35.5 to 44.2)	39.7 (34.5 to 44.9)	−0.1 (−6.9 to 6.7)
≥75,000	33.5** (31.1 to 35.9)	23.9** (20.6 to 27.3)	−9.6 (−13.7 to −5.5)
**Region**			
Northeast (ref)	35.2 (31.3 to 39.1)	35.5 (29.6 to 41.4)	0.3 (−6.8 to 7.4)
Midwest	36.7 (33.0 to 40.4)	30.3 (25.3 to 35.3)	−6.4 (−12.6 to −0.2)
South	41.1** (38.3 to 44.0)	32.8 (27.5 to 38.2)	−8.3 (−14.4 to −2.2)
West	36.7 (33.2 to 40.1)	29.9 (24.4 to 35.4)	−6.8 (−13.3 to −0.3)
**Health insurance status**			
Private health insurance (ref)	37.8 (35.6 to 40.0)	30.7 (27.2 to 34.3)	−7.1 (−11.3 to −2.9)
Public health insurance	35.3 (32.4 to 38.2)	29.6 (25.1 to 34.2)	−5.7 (−11.1 to −0.3)
No health insurance	48.7** (42.1 to 55.2)	44.5** (33.4 to 55.5)	−4.2 (−17.0–8.6)
**MSA status**			
Metro (ref)	36.9 (35.1 to 38.7)	30.2 (27.0 to 33.4)	−6.7 (−10.4 to −3.0)
Nonmetro	46.2** (41.3 to 51.1)	39.6** (33.5 to 45.7)	−6.6 (−14.4 to 1.2)
**2020–21 influenza vaccination status**			
Received influenza vaccination/Absolutely certain (ref)	23.3 (21.2 to 25.5)	14.7 (12.0 to 17.3)	−8.6 (−12.0 to −5.2)
Very likely/Somewhat likely	30.3** (27.0 to 33.6)	20.6 (14.6 to 26.5)	−9.7 (−16.5 to −2.9)
Not likely	67.0** (63.9 to 70.2)	68.3** (63.7 to 72.9)	1.3 (−4.3–6.9)
**Concern about COVID-19 illness for self**			
Very/Somewhat concerned (ref)	27.6 (25.6 to 29.8)	18.8 (15.9 to 21.7)	−8.8 (−12.4 to −5.2)
Slightly/Not concerned	50.1** (47.4 to 52.7)	51.3** (47.2 to 55.3)	1.2 (−3.6 to 6.0)
**Concern about side effects of vaccine for self**			
Very/Somewhat concerned (ref)	43.7 (41.5 to 46.0)	40.5 (36.7 to 44.2)	−3.2 (−7.6 to 1.2)
Slightly/Not concerned	28.9** (26.3 to 31.6)	21.5** (18.4 to 24.6)	−7.4 (−11.5 to −3.3)
**Trust governmental approval process to ensure the COVID-19 vaccine is safe for the public**			
Fully/Mostly trust (ref)	9.5 (7.9 to 11.2)	7.7 (5.6 to 9.9)	−1.8 (−4.5 to 0.9)
Somewhat trust/Do not trust	56.7** (54.4 to 58.9)	54.3 (50.4 to 58.2)	−2.4 (−6.9 to 2.1)

**Abbreviations:** CI = confidence interval; COVID-19 = coronavirus disease 2019; MSA = metropolitan statistical area; ref = reference category.

* IPSOS KnowledgePanel Survey, fielded September 3–October 1.

^†^ IPSOS KnowledgePanel Omnibus Survey, fielded December 18–20.

^§^ NORC AmeriSpeak Omnibus Survey, fielded December 18–20.

^¶^ CIs for differences that exclude zero are statistically significant.

** p<0.05 compared with respective reference category for each variable (by t-test).

Among adults in the December surveys who did not intend to get vaccinated, the main reasons most frequently cited were concerns about side effects and safety of the COVID-19 vaccine (29.8%), planning to wait to see if the vaccine is safe and consider receiving it later (14.5%), lack of trust in the government (12.5%), and concern that COVID-19 vaccines were developed too quickly (10.4%) (Table 3). A larger percentage of the December survey participants than September participants reported safety concerns as a main reason (29.8% versus 23.4%), and a smaller percentage reported concern that vaccines were developed too quickly (10.4% versus 21.6%).

**TABLE 3:** Main reasons for not intending to get COVID-19 vaccine,* United States, September and December 2020

Main reasons	Weighted % (95% CI)		
	IPSOS, Sep 2020^†^ (n = 3,541)	Average of Dec IPSOS^§^ and NORC^¶^ estimates (n = 2,033)	Difference between Dec and Sep estimates**
Concern about the side effects and safety of the vaccine	23.4 (20.9 to 25.9)	29.8 (26.2 to 33.4)	6.4 (2.0 to 10.8)
Concern that the vaccine is being developed too quickly	21.6 (19.3 to 24.1)	10.4 (7.6 to 13.2)	−11.2 (−14.9 to −7.5)
Plan to wait and see if it is safe and may get it later	18.0 (15.7 to 20.2)	14.5 (11.1 to 17.9)	−3.5 (−7.6 to 0.6)
Don’t trust the government	9.8 (8.0 to 11.6)	12.5 (9.0 to 15.9)	2.7 (−1.2 to 6.6)
Plan to use masks/other precautions instead	3.4 (2.4 to 4.4)	3.7 (1.4 to 6.0)	0.3 (−2.2 to 2.8)
Don’t like vaccines	3.2 (2.2 to 4.1)	5.4 (3.0 to 7.9)	2.2 (−0.4 to 4.8)
Not a member of any group that is at high risk for COVID-19	2.8 (1.9 to 3.8)	3.5 (1.8 to 5.1)	0.7 (−1.2 to 2.6)
COVID-19 is not a serious illness	2.6 (1.6 to 3.6)	1.9 (0.8 to 3.0)	−0.7 (−2.2 to 0.8)
The vaccine will not work	2.4 (1.5 to 3.3)	0.0 (—)	−2.4 (−3.3 to −1.5)
The vaccine could give me COVID-19	2.4 (1.5 to 3.3)	2.3 (0.0 to 5.4)	−0.1 (−2.9 to 2.7)
Had COVID-19 and should be immune	1.0 (0.4 to 1.6)	2.2 (1.0 to 3.5)	1.2 (−0.2 to 2.6)
Don’t like needles	1.0 (0.5 to 1.6)	3.0 (0.1 to 6.0)	2.0 (−1.0 to 5.0)
Doctor has not recommended a COVID-19 vaccine to me	0.8 (0.4 to 1.4)	0.0 (—)	−0.8 (−1.3 to −0.3)
Didn’t know I needed a vaccine against COVID-19	0.2 (0.0 to 0.5)	0.4 (0.0 to 1.0)	0.2 (−0.4 to 0.8)
Concern about the costs associated with the vaccine (such as office visit costs or vaccine administration fees)	0.2 (0.0 to 0.3)	0.2 (0.0 to 0.8)	0.0 (−0.4 to 0.4)

**Abbreviations:** CI = confidence interval; COVID-19 = coronavirus disease 2019.

* Among respondents who stated that they are not likely to receive the COVID-19 vaccine.

^†^ IPSOS KnowledgePanel Survey, fielded September 3–October 1.

^§^ IPSOS KnowledgePanel Omnibus Survey, fielded December 18–20.

^¶^ NORC AmeriSpeak Omnibus Survey, fielded December 18–20.

^**^ CIs for differences that exclude zero are statistically significant.

## Discussion

From September to December 2020, vaccination intent increased among all adults by approximately 10 percentage points and across all priority groups, with the largest increase in intent to be vaccinated among adults aged ≥65 years; vaccination nonintent decreased among all adults by 6 percentage points and across most sociodemographic groups. However, despite increases in vaccination intent since September (*5*), only about half of persons aged 18–64 years surveyed in December reported being very likely to receive COVID-19 vaccination, even among those who were essential workers and persons aged 18–64 years with underlying medical conditions. Younger adults, women, Black persons, adults living in nonmetropolitan areas, and adults with lower educational attainment, with lower income, and without insurance were most likely to report that they did not intend to receive COVID-19 vaccination. Several studies found similar percentages and trends in vaccination intent and low likelihood of receiving a COVID-19 vaccine among groups disproportionately affected by COVID-19, including Black persons and those with lower educational attainment (*6*,*7*). Because many of these groups are at increased risk for COVID-19–associated morbidity and mortality (*8*), COVID-19 vaccination is important for protecting the health of these populations and reducing health inequities.

The findings in this report are subject to at least seven limitations. First, although panel recruitment methodology and data weighting were designed to produce nationally representative results, respondents might not be fully representative of the general U.S. adult population. Second, because the sample of persons surveyed in December was not derived from the sample of persons surveyed in September, longitudinal analysis of changes in perception from the same sample of persons was not possible. Third, small sample sizes prevented separate analyses of some priority groups identified by ACIP, such as health care personnel, frontline and other essential workers, and adults aged 65–74 years and ≥75 years. Fourth, because essential worker status and high-risk medical conditions were self-reported, there might be potential for misclassification. Respondents were also placed into mutually exclusive vaccine priority groups, which could not account for persons who fit within multiple groups (e.g., essential workers aged 18–64 years with underlying medical conditions). Fifth, attitudes and perceptions might change quickly, and these results might not be reflective of current reasons for not intending to receive a COVID-19 vaccine. Sixth, results are national estimates and cannot be generalized to the state or local level. Finally, results might not be comparable to other national polls or surveys because of potential differences in survey methods, sample population, and questions related to vaccination intent.

Continuing to promote vaccine confidence by tailoring information to address concerns of individual persons and communities is critical to preventing the spread of COVID-19. These findings suggest a decrease in nonintent over time as well as concerns about vaccine safety among priority populations in the United States and have implications for potential messages and strategies that could boost confidence in COVID-19 vaccines and educate essential workers, minority populations, and the general public about the safety of the vaccine development process, and the known effectiveness and safety of authorized COVID-19 vaccines (*9*). Health care providers are known to be a trusted source of information about vaccines for many persons and can use CDC-recommended guidance to have effective conversations with patients about the need for vaccination (*10*). Ensuring high and equitable vaccination coverage in all populations is critical to preventing the spread of COVID-19 and bringing an end to the pandemic.

**Summary**

**What is already known about this topic?**

National polls conducted before vaccine distribution began suggested that many persons were hesitant to receive COVID-19 vaccination.

**What is added by this report?**

From September to December 2020, intent to receive COVID-19 vaccination increased from 39.4% to 49.1% among adults and across all priority groups, and nonintent decreased from 38.1% to 32.1%. Despite decreases in nonintent from September to December, younger adults, women, non-Hispanic Black adults, adults living in nonmetropolitan areas, and adults with less education and income, and without health insurance continue to have the highest estimates of nonintent to receive COVID-19 vaccination.

**What are the implications for public health practice?**

Ensuring high and equitable vaccination coverage among all populations, including by addressing reasons for not intending to receive vaccination, is critical to prevent the spread of COVID-19 and bring an end to the pandemic.

## Footnotes

* Persons with underlying medical conditions were defined as those who reported having any of the following conditions: cancer; chronic kidney disease; chronic obstructive pulmonary disease (COPD); heart conditions (e.g., heart failure, coronary artery disease, or cardiomyopathies); immunocompromised state (weakened immune system) from solid organ transplant; obesity; pregnancy; sickle cell disease; smoking; and type 2 diabetes mellitus. Respondents aged 18–64 years reporting diagnosis of one or more of these conditions were classified as high-risk in the analyses. This list of underlying medical conditions does not include Down syndrome, which was added to the list on December 23, 2020. A complete list of underlying medical conditions is available at https://www.cdc.gov/coronavirus/2019-ncov/need-extra-precautions/people-with-medical-conditions.html

† Other essential workers include those who conduct a range of operations and services that are essential to continued critical infrastructure viability, including staffing operations centers, maintaining and repairing critical infrastructure, operating call centers, working construction, and performing operational functions, among others. Also included are workers who support crucial supply chains and enable functions for critical infrastructure. The industries they support represent, but are not limited to, medical and health care, telecommunications, information technology systems, defense, food and agriculture, transportation and logistics, energy, water and wastewater, and law enforcement.

§
https://www.ipsos.com/sites/default/files/ipsosknowledgepanelmethodology.pdf

¶
https://www.ipsos.com/en-us/solutions/public-affairs/knowledgepanel-omnibus

**
https://amerispeak.norc.org/our-capabilities/Pages/AmeriSpeak-Omnibus.aspx

†† The panels from the September and December surveys use an address-based sampling methodology that covers nearly all households in the United States regardless of their phone or Internet status, with a cooperation rate (proportion of all cases interviewed among all eligible units ever contacted) of 69.7% (September IPSOS survey), 38.0% (December IPSOS survey), and 22.8% (December NORC survey). Surveys were fielded in English and Spanish, and non-Hispanic Black and non-Hispanic other race panel members were oversampled to ensure adequate sample size for subgroup analyses by respondent’s race/ethnicity.

§§ 45 C.F.R. part 46.102(l)(2), 21 C.F.R. part 56; 42 U.S.C. Sect. 241(d); 5 U.S.C. Sect. 552a; 44 U.S.C. Sect. 3501 et seq.

¶¶ Essential workers were defined as those who responded “yes” to the following question: “In your work or volunteer activities, are you classified as an essential worker?”

*** Mutually exclusive groups were categorized in the following order: essential workers, adults aged ≥65 years, and adults aged 18–64 years with an underlying medical condition. Anyone who self-identified as an essential worker was categorized as an essential worker, regardless of age. Next, anyone aged ≥65 years was categorized as adult aged ≥65 years. Finally, anyone aged 18–64 years with an underlying medical condition was categorized as adult aged 18–64 years with an underlying medical condition. All others were categorized as adults aged 18–64 years who were not essential workers and had no underlying medical conditions.

††† Metropolitan statistical area (MSA) status was determined by census block group using the panelist’s address. For a small number of panelists for whom the address was not available, ZIP code was used to determine MSA status. https://www.census.gov/programs-surveys/metro-micro.html
